# Co-seismic landslide topographic analysis based on multi-temporal DEM—A case study of the Wenchuan earthquake

**DOI:** 10.1186/2193-1801-2-544

**Published:** 2013-10-17

**Authors:** Zhikun Ren, Zhuqi Zhang, Fuchu Dai, Jinhui Yin, Huiping Zhang

**Affiliations:** State Key Laboratory of Earthquake Dynamics, Institute of Geology, China Earthquake Administration, Beijing, 100029 China; Institute of Geology and Geophysics, Chinese Academy of Sciences, P.O. Box 9825, Beijing, 100029 P.R. China

**Keywords:** Co-seismic landslide, Topographic, PGA, Multi-temporal DEM, Wenchuan earthquake

## Abstract

Hillslope instability has been thought to be one of the most important factors for landslide susceptibility. In this study, we apply geomorphic analysis using multi-temporal DEM data and shake intensity analysis to evaluate the topographic characteristics of the landslide areas. There are many geomorphologic analysis methods such as roughness, slope aspect, which are also as useful as slope analysis. The analyses indicate that most of the co-seismic landslides occurred in regions with roughness, hillslope and slope aspect of >1.2, >30, and between 90 and 270, respectively. However, the intersection regions from the above three methods are more accurate than that derived by applying single topographic analysis method. The ground motion data indicates that the co-seismic landslides mainly occurred on the hanging wall side of Longmen Shan Thrust Belt within the up-down and horizontal peak ground acceleration (PGA) contour of 150 PGA and 200 gal, respectively. The comparisons of pre- and post-earthquake DEM data indicate that the medium roughness and slope increased, the roughest and steepest regions decreased after the Wenchuan earthquake. However, slope aspects did not even change. Our results indicate that co-seismic landslides mainly occurred at specific regions of high roughness, southward and steep sloping areas under strong ground motion. Co-seismic landslides significantly modified the local topography, especially the hillslope and roughness. The roughest relief and steepest slope are significantly smoothed; however, the medium relief and slope become rougher and steeper, respectively.

**Electronic supplementary material:**

The online version of this article (doi:10.1186/2193-1801-2-544) contains supplementary material, which is available to authorized users.

## Introduction

It has been commonly accepted that steep topography are of high landslide frequency in active orogenic region. The co-seismic landslides usually occurred in active orogenic regions, which are one of the major secondary nature hazards related to strong earthquakes (Harp and Jibson 1996; Gallousi and Koukouvelas 2007; Owen et al. 2008; Ren and Lin 2010; Dai et al. 2011a). In some cases, co-seismic landslides even produce more serious human loss and damages than the earthquake itself. Thus, the co-seismic landslides have fundamental influence on human life and seismic design of buildings etc. It has been noticed that transportation and deposition of the landslide materials will also have fundamental impact on the topographic evolution (Meng et al. 2006; Godard et al. 2010; Ouimet, 2010; Hovius et al. 2011; Parker et al. 2011). Contemporary, the topographic conditions will also affect the susceptibility of landslides (Jibson et al. 2000; Dai and Lee 2002; Korup et al. 2007). A variety of approaches have been used in slope instability analysis, which has been one of the most important topographic features in detecting susceptible landslide areas (Dai and Lee 2002; Korup et al. 2007; Ren and Lin 2010; Chuang and Fabbri 2008). With the development of Geographical Information Systems (GIS), numerous quantitative topographic analysis approaches have developed in recent years. Topographic roughness, slope aspect and hillslope are the most commonly used features in tectonic geomorphologic and landslide-related studies (e.g., Dai and Lee 2002; Casson et al. 2005; Zhang et al. 2011; Ren and Lin 2010). The lithologic units and concentrations of the co-seismic landslides induced by Wenchuan earthquake have been analyzed in detail (Dai et al. 2011a). In this paper, we will mainly focus on the topographic characteristics of the co-seismic landslides. The shaking intensity is another important parameter that has been thought to be related to co-seismic landslides as well as sand liquefactions (Harp and Wilson 1995; Murphy et al. 2002; Meunier et al. 2007; Wang et al. 2011). In this study, we also use the open accessed strong motion records to analyze the relationship between Wenchuan earthquake triggered co-seismic landslides and the peak ground acceleration (PGA) distribution, which is distributed by CSMNC (China Strong Motion Networks Center), IEMCEA (Institute of Engineering Mechanics, China Earthquake Administration).

The occurrence of 2008 Mw 7.9 Wenchuan earthquake provides a valuable opportunity to verify the accuracy of multiple approach analyses involving variable topographic analyses and shaking intensity. The landslide areas are validated using the pre-earthquake high-resolution digital elevation model (DEM) data derived from 1:50,000 topographic maps. The analyses indicate that the co-seismic landslides are closely correlated to the topographic conditions as well as the shaking intensity. The topographic effects of the Wenchuan earthquake are also analyzed based on post-earthquake DEM data derived from stereo pair of IRS-P5 (Indian Remote Sensing Satellite) remote sensing images by comparing with pre-earthquake DEM.

## Study area

The Longmen Shan region is one of the steepest margins of the Tibetan Plateau accompany with almost 5000 m altitude decrease across the margin within 50 km distance (Burchfiel et al. 1995, 2008; Chen and Wilson 1996, Figure 1). It is also an active orogen whose activity has been largely underestimated due to the less crustal shortening and low slip rates of the major faults within it, as revealed by low geodetic slip rates, shortening rates (King et al. 1997; Chen et al. 2000; Zhang et al. 2004; Gan et al. 2007) and geological slip rates (Densmore et al. 2007; Zhou et al. 2007). There are four major active fault systems within the Longmen Shan Thrust Belt: Wenchuan-Maowen, Yingxiu-Beichuan, Guanxian-Anxian and Qingchuan fault systems (Burchfiel et al. 1995, 2008; Densmore et al. 2007; Zhou et al. 2007). It has been reported that the Wenchuan earthquake ruptured the Yingxiu-Beichuan and Guanxian-Anxiang faults (Lin et al. 2009; Xu et al. 2009; Liu-Zeng et al. 2009; Zhang et al. 2010), although it is still ambiguous that whether the Qingchuan fault is ruptured (Lin et al. 2012). A large amount of co-seismic landslides were triggered on the steep slope regions accompany with the co-seismic deformations (e.g. Ren and Lin, 2010; Yin et al. 2010; Dai et al. 2011a,2011b; Parker et al. 2011; Tang et al. 2011), which significantly changed the local topography. The co-seismic landslides mainly distributed in a corridor that bounds the co-seismic surface rupture, with major portion on the hanging wall side (Ren and Lin 2010; Yin et al. 2010; Godard et al. 2010; Ouimet 2010; Dai et al. 2011a, 2011b). Thus, the earthquake triggered deformation and landslides can dominate local erosion and landscape evolution in Longmen Shan region (Meng et al. 2006), as in other active orogen in the world (Hovius et al. 2011; Mackey and Roering 2011). Therefore, the co-seismic landslides of the Wenchuan earthquake should be controlled by the topographic conditions and will consequently affect the local topographic evolution in Longmen Shan region.

**Figure 1:**
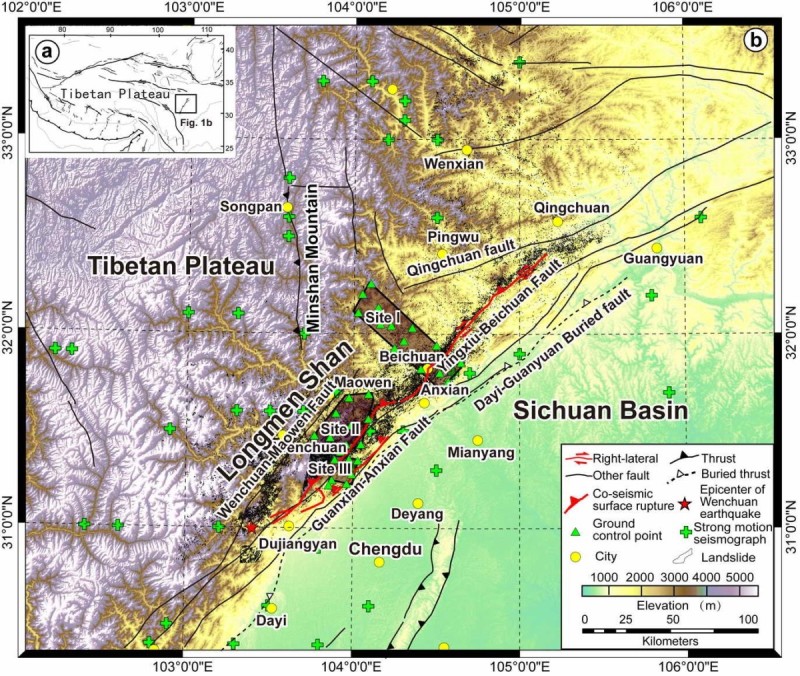
**Simplified tectonic map of the Tibetan Plateau (a) and Longmen Shan region (b).** Tectonic structures are modified from (Lin, et al. 2009, 2012 Liu-Zeng et al. 2009 Xu et al. 2009 Zhang et al. 2010). Thick red lines on the main map indicate the co-seismic surface ruptures of the 2008 Mw 7.9 Wenchuan earthquake. Green crosses indicate the strong motion seismograph stations. Green triangles indicate the GPS survey locations for ground control points to improve the quality of DEM data derived from stereo pairs of remote sensing imagery data. Black polygons show the co-seismic landslides. Translucent polygons indicate the study areas of Site I, II and III.

## Data and methods

### Data acquirement

With the development of GIS techniques, recent quantitative topographic analyses are usually based on DEM data. Previous studies suggest that the DEM data derived from stereo pair of remote sensing imagery data is favorable in landslide detection (Casson et al. 2005). The Indian Remote Sensing Satellite P5 (IRS-P5) carries two Panchromatic cameras (PAN fore and PAN after), which is favorable for deriving post-earthquake DEM. In this study, we derived a 5 m resolution DEM using the stereo pairs of 2.5 m resolution IRS-P5 imagery data. Three representative sites (Site I-III) were selected for DEM derivation, which have no cloud coverage at the time of imagery acquisition (Table 1). To improve the DEM quality, we also applied field ground control points (GCP) survey using Trimble R8 GPS (Figure 1). Finally, we derived the DEM using the GCP points, Rational Polynomial Coefficient (RPC) camera model (Tao and Hu 2001) and tie points between the Pan fore and Pan after imagery. The checkpoints indicate that the error of the post-earthquake DEM is usually less than 1.5 m (Tables 2 and 3), which is precise enough for landslide topography analysis. Because there are no pre-earthquake stereo pairs of remote sensing imagery data available, the pre-earthquake DEM data are derived from the best data we can get—the 1:50,000 topographic maps. The topographic maps are surveyed using stereo pairs of aerial photograph with sufficient ground control points (GCP) by National Administration of Surveying, Mapping and Geoinformation of China (NASMGC) in 1970s.

**Table 1:** **Stereo pairs IRS-P5 Imagery used in post-earthquake DEM derivation**

No.	Path	Row	Date
1	659	250	20080603
2	659	251	20080603
3	659	252	20080603
4	659	253	20080603
5	659	254	20080603

**Table 2:** **Elevation precision of topographic map derived pre-earthquake DEM**

No.	Longitude(°)	Latitude(°)	GPS_Elevation(m)	DEM_Elevation(m)	Error(m)
1	106.023	32.9621	616.749	620.08234	3.3333364
2	105.457	32.0184	678.851	682.28235	3.4313486
3	103.435	32.9307	3587.7	3583.9824	-3.7175781
4	102.501	32.7856	3471.93	3462.3823	-9.5476758
5	104.571	32.4053	1312.77	1312.4823	-0.2877002
6	104.831	32.1819	1068.06	1064.7823	-3.2776514
7	104.444	31.8019	1002.18	1002.1824	0.0023731
8	103.166	32.075	2957.83	2948.6824	-9.1476269
9	104.782	31.4865	489.381	484.88232	-4.4986758
10	104.187	31.3529	571.82	571.2334	-0.5866016
11	104.441	31.1571	483.26	484.68231	1.422312
12	104.077	30.732	490.226	494.68231	4.456312
13	103.41	30.415	559.666	550.9823	-8.6837002
14	106.034	30.8042	295.216	299.77786	4.5618625

The mean elevation error is calculated to be ±4.1 m.

**Table 3:** **Elevation precision of imagery derived post-earthquake DEM**

No.	Longitude(°)	Latitude(°)	GPS_Elevation(m)	DEM-Elevation(m)	Error(m)
1	103.89948	31.34582	1390.878	1393.403	-2.525
2	103.96341	31.22905	876.761	874.376	2.385
3	104.02967	31.34853	798.949	799.126	-0.177
4	103.99306	31.27809	738.503	737.37	1.133
5	103.9768	31.35489	1090.177	1090.542	-0.365
6	104.53361	31.87152	599.059	598.938	0.121
7	104.22619	32.03414	1104.028	1103.25	0.778
8	104.35132	31.95228	1470.297	1467.909	2.388
9	104.4132	31.8329	942.5816	940.137	2.4446
10	104.58483	31.82802	571.4206	572.47	-1.0494
11	104.52359	31.778	806.9447	805.641	1.3037
12	103.942	31.31536	942.3366	946.256	-3.9194

The elevation mean error is calculated to be ±1.5 m.

Previous DEM precision check tables show the mean error of the pre-earthquake DEM is ±4.1 m, and median error is ±3.6 m; the mean error of the post-earthquake DEM is ±1.5 m, and mean elevation error is ±1.2 m.

### Methods

#### Topographic analyses

##### Roughness

**Figure 2:**
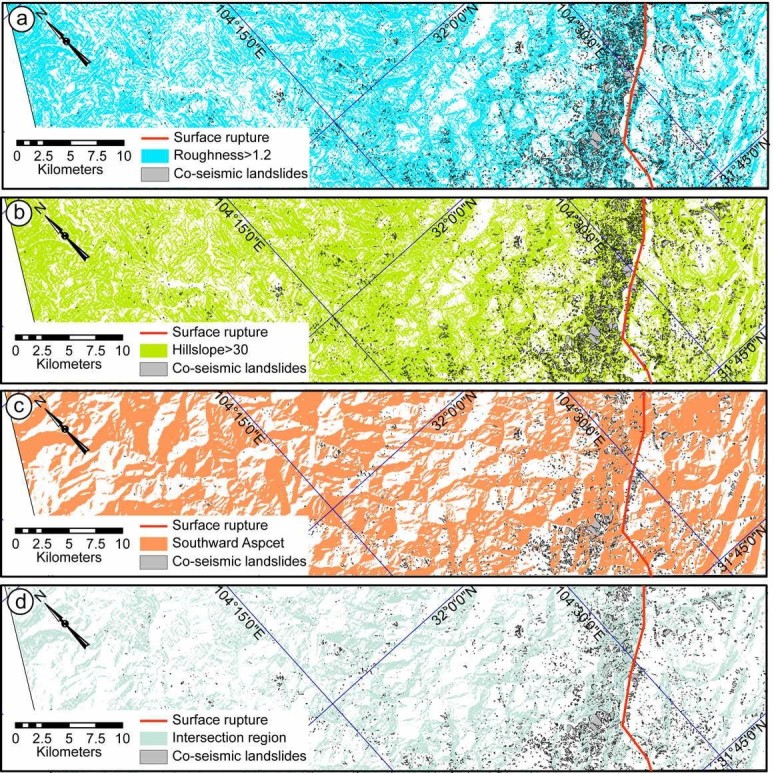
**Topographic features of Site I.** The roughness **(a)**, hillslope **(b)**, slope aspect **(c)** distribution of Site I based on pre-earthquake DEM data; and the intersection region of the above three regions **(d)** by applying thredholds >1.2 **(a)**, >30 **(b)** and between 90 and 270 **(c)**. The maps show the landslide mainly occurred in the derived regions.

**Figure 3:**
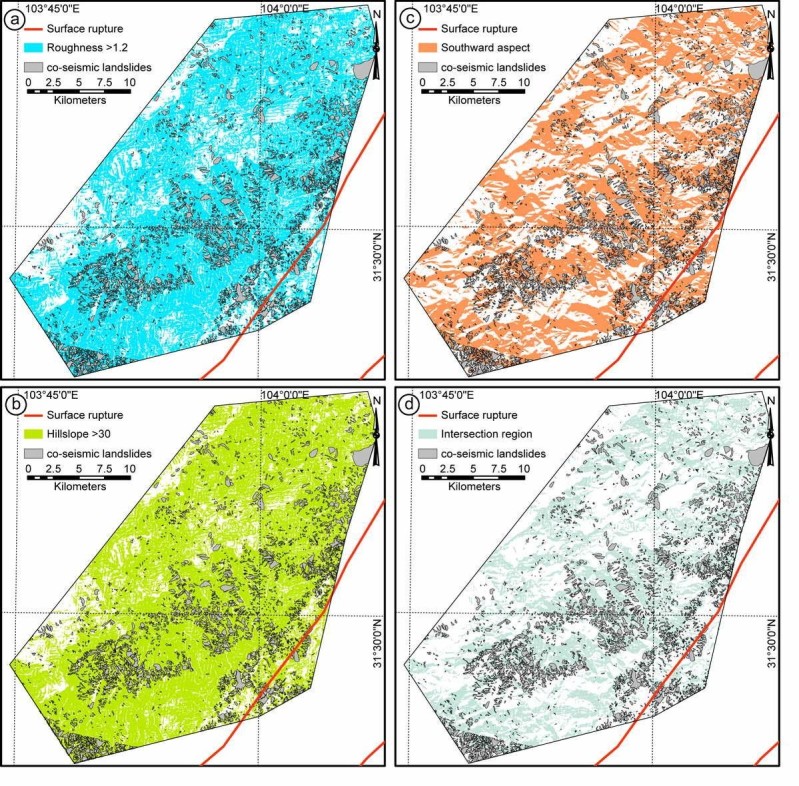
**Topographic features of Site II.** The roughness **(a)**, hillslope **(b)**, slope aspect **(c)** distribution of Site II based on pre-earthquake DEM data; and the intersection region of the above three regions **(d)** by applying thresholds >1.2, between 90 and 270, and 30.

**Figure 4:**
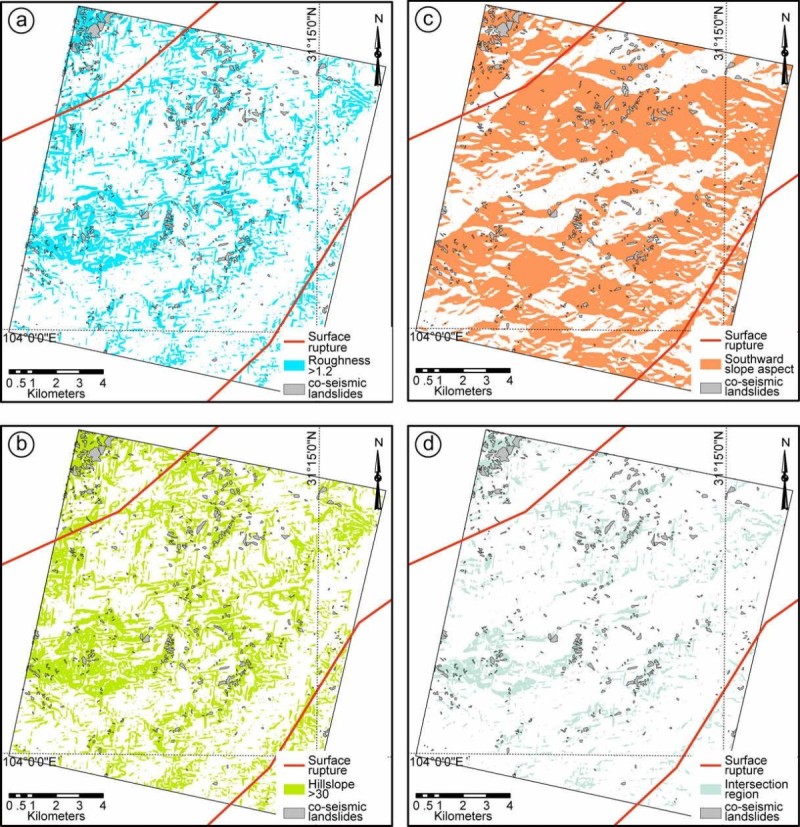
**Topographic features of Site III.** The roughness **(a)**, hillslope **(b)**, slope aspect **(c)** distribution of Site III based on pre-earthquake DEM data; and the intersection region of the above three regions **(d)** by applying thresholds >1.2, between 90 and 270, and 30.

**Figure 5:**
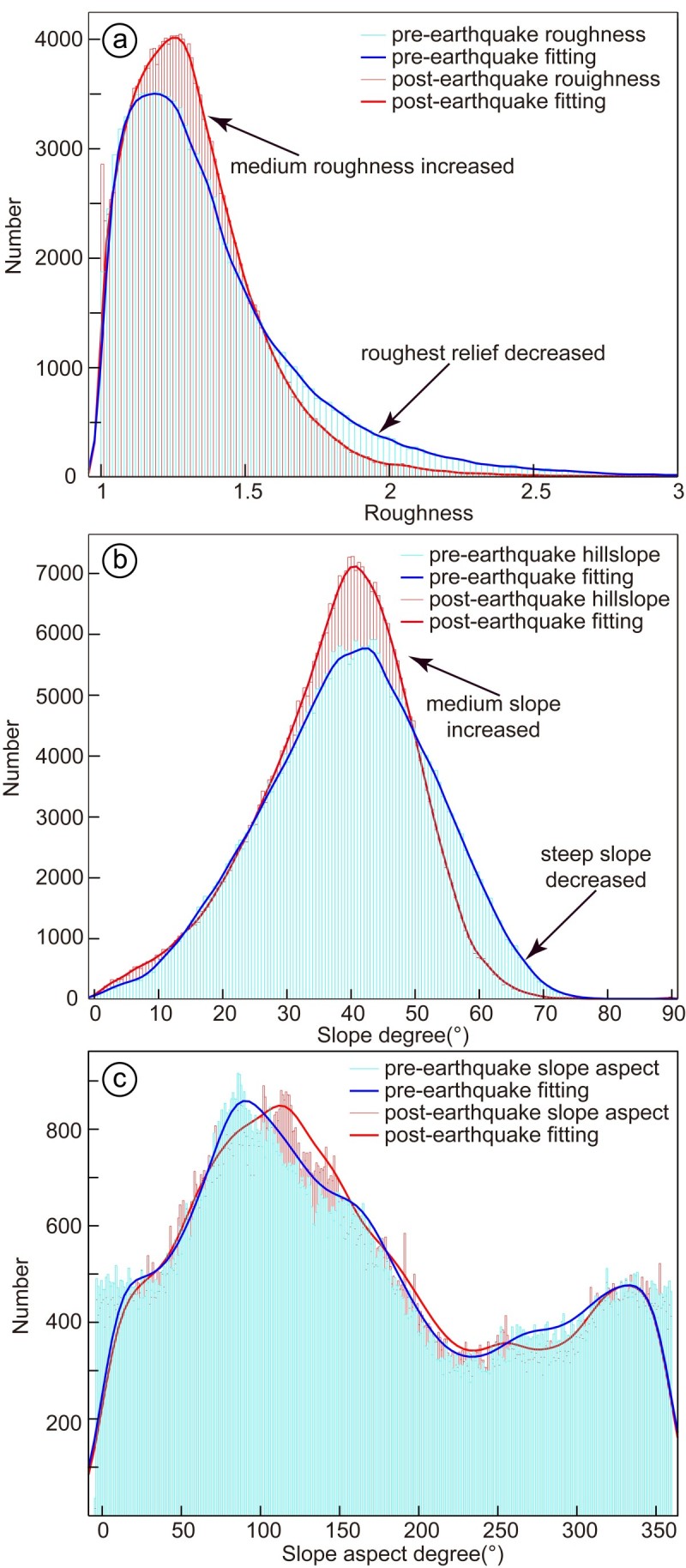
**Co-seismic topographic changes.** Distribution of Pre-earthquake and post-earthquake hillslope **(a)**, roughness **(b)** and slope aspect **(c)**. The medium topography are roughened and steepened, however the steepest and roughest topography are smoothed by the Wenchuan earthquake. The slope aspect is almost unchanged.

To express the ragged topography, roughness is a widely-used factor in topographic studies. Roughness is defined by the ratio between the surface area and projected area. It is usually obtained by averaging the roughness value over an area of 3 × 3 pixel size. By running a test to determine the best threshold on the balance of the percentage of landslides within the derived region and corresponding area, the threshold of 1.2 for roughness could be determined (Figures 2, 3 and 4). It shows that most of the landslides occurred in regions with roughness larger than 1.2, i.e., the threshold of 1.2 is the most effective value in landslide detection. We also compared the pre- and post-earthquake roughness within the landslide area. It indicates that the medium topographic roughness significantly increased after earthquake. The roughest relief is also smoothed by the Wenchuan earthquake (Figure 5(b)).

##### Hillslope

Slope instability analysis has been one of the most important topographic analyses regarding landslide susceptibility. It has been testified that steep slope areas are prone to landslides (Burbank et al. 1996; Densmore et al. 1998; Dai and Lee 2002). The co-seismic landslides of the Wenchuan earthquake mainly distribute in regions with slopes larger than 30° on pre-earthquake DEM (Ren and Lin 2010; Figures 2, 3 and 4). Thus, we analysis the relationship between the co-seismic landslides and slope distribution by applying a threshold of 30° on the slope distribution map (Figures 2, 3 and 4, Table 4). In order to detect the slope changes produced by the co-seismic landslides, we compared the slope distribution pre- and post-earthquake within the landslide area. It indicates that the medium slope significantly increased after earthquake, which indicates that the topography is becoming steeper. However, the steepest slopes decreased after earthquake, which indicates that the Wenchuan earthquake also smoothed the pre-earthquake steepest slopes (Figure 5(a)).

**Table 4:** **Topographic analysis of the co-seismic landslides produced by the 2008 Wenchuan earthquake**

	LD	IAD	EALD
Site I			
Slope aspect	51.73%	45.46%	23.52%
Roughness	56.72%	54.10%	30.68%
Slope	67.33%	41.85%	28.18%
Intersection	30.55%	100%	30.55%
Site II			
Slope aspect	50.34%	69.31%	34.89%
Roughness	75.96%	53.89%	40.94%
Slope	82.85%	47.46%	39.32%
Intersection	38.34%	100%	38.34%
Site III			
Slope aspect	66.50%	20.77%	13.81%
Roughness	46.90%	56.57%	33.04%
Slope	58.42%	36.79%	17.25%
Intersection	34.14%	100%	34.14%

LD: Landslide density: landslides occurred within the derived regions based on topographic analysis of roughness, hill slope and slope aspect by applying corresponding thresholds (detail thresholds refer to the text); IAD: intersection area density, represents the area percentage of the intersection area within the derived regions; EALD: Equal area landslide density, by multiple the landslide percentage with the intersection area percentage which is used to describe the efficiency of each method in landslide occurrence detection.

##### Slope aspect

The slope aspect is the expression of horizontal direction that a mountain slope faces. Due to the exposure to sunrays, the slope aspect has fundamental influence on the landslide possibility due to the differences of temperature, sediment condition, vegetation etc. (e.g. Rech et al. 2001; Fekedulegn et al. 2003). Mechanical, chemical and biological weathering are much stronger on the southward facing slopes than that on the northward facing slopes, where are more open to the sunlight and warm wind. The slope aspect analysis on post-earthquake DEM also shows high landslide density on the southward facing slopes (Figures 2, 3 and 4). The southward slope aspect is generally between 90 and 270 degree. Therefore, applying threshold value of 90—270, we can derive the landslide potential area on the basis of the slope aspect analysis. According to the slope aspect, post-earthquake slope aspect did not change much, comparing with pre-earthquake data within the landslide area. It indicates that the co-seismic landslides did not affect the slope aspect, i.e., it is not controlled by such tectonic events.

#### Seismic information

According to the co-seismic landslides, seismic information such as ground motion is one of the main trigger mechanisms. We consequently analyzed the characteristics of landslide areas based on multiple approaches including ground motion information.

**Figure 6:**
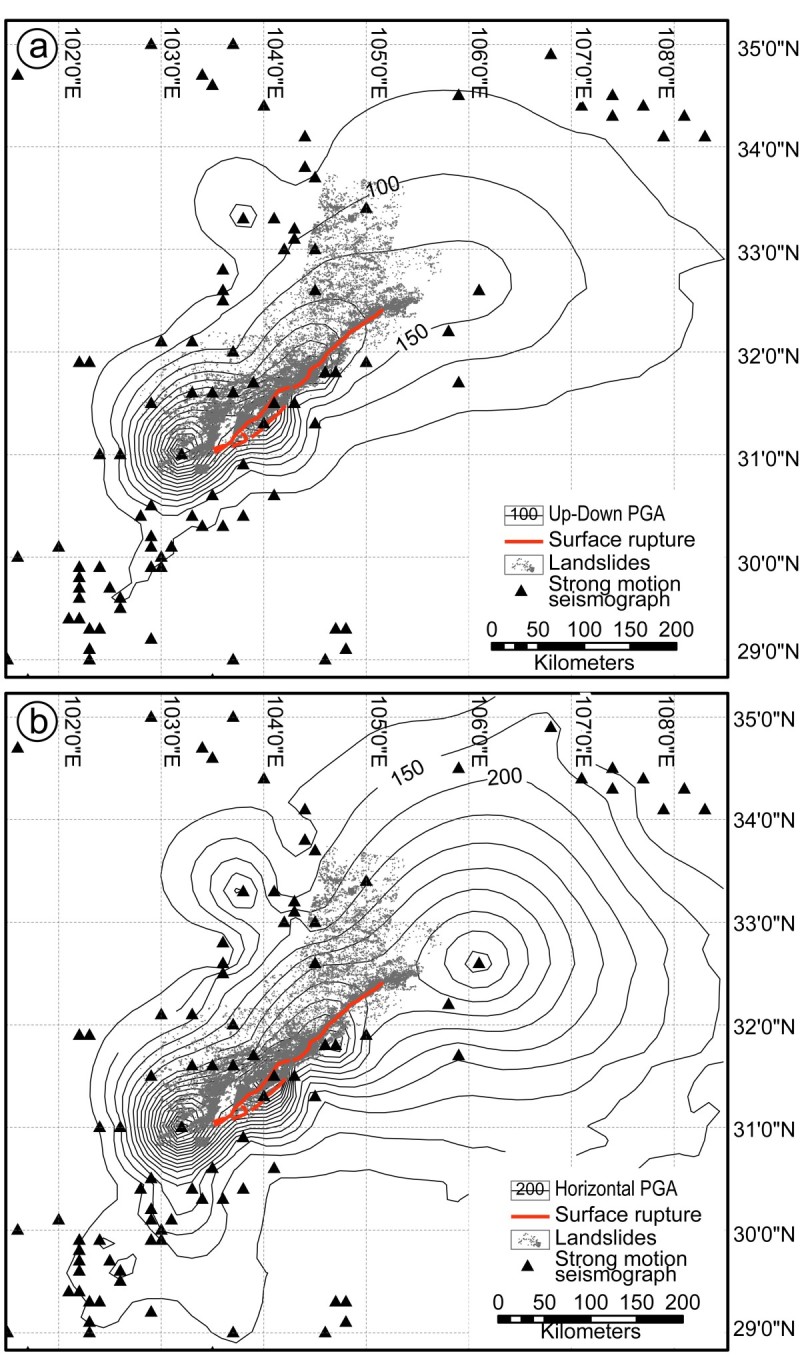
Relationship between PGA and distribution of co-seismic landslides. Up-down PGA **(a)** and horizontal PGA **(b)** contours with the distribution of co-seismic landslides produced by the Wenchuan earthquake.

Preliminary analyses of the strong motion records show that the peak ground acceleration (PGA) contours distribute in the region peripheral to the co-seismic surface rupture with the maximum value of 957.7 gal (Li et al. 2008; Figure 6). We collected the strong motion records of 267 stations in the regions affected by Wenchuan earthquake. The PGA maps of the UD (up-down) and horizontal (combination of north–south and east–west) vectors, respectively, show close relation with the co-seismic surface rupture zone (Figure 6(a) and (b)). Previous studies have demonstrated that PGA also could be used as a threshold to evaluate landslide susceptibility (Harp and Wilson 1995; Murphy et al. 2002; Meunier et al. 2007; Wang et al. 2011). Recent study suggests that the co-seismic landslide of the Wenchuan earthquake is closely related to the PGA value (Wang et al. 2011). The co-seismic landslides are also directly controlled by the geological structures such as the co-seismic surface ruptures. Previous study has demonstrate the significant hanging wall effect (Ren and Lin 2010), thus according to thrust fault, landslide occurrence will mainly occupy the hanging wall side. The co-seismic landslides triggered by the Wenchuan earthquake mainly occurred on the hanging wall side where confined by the up-down and horizontal PGA contour of 150 gal and 200 gal, respectively (Figure 6(a) and (b)).

## Discussion

Co-seismic landslide is a kind of special landslide directly triggered by strong earthquake (Harp and Jibson 1996; Gallousi and Koukouvelas 2007; Owen et al. 2008; Ren and Lin 2010; Dai et al. 2011a). Therefore, the co-seismic landslides are related to the topographic situation and seismic shaking. In landslide-related studies, roughness, hillslope and slope aspect analyses are the most widely used methods in geomorphologic studies (Dai and Lee 2002; Casson et al. 2005; Ren and Lin 2010; Korup et al. 2007; Chuang and Fabbri 2008). After the 2008 Mw 7.9 Wenchuan earthquake, there are numerous co-seismic landslides were triggered (Ren and Lin 2010; Yin et al. 2010 Godard et al. 2010; Ouimet 2010; Dai et al. 2011a, 2011b), which provide an ideal opportunity to check the co-seismic landslides characters. Based on the pre-earthquake DEM data, we can analysis the topographic character of the landslided area. Comparing pre-earthquake and post-earthquake DEM data, we can consequently analysis the topographic effects of the Wenchuan earthquake. In order to derive the high landslide density region, the threshold values of roughness, hillslope and slope aspect are set to be >1.2, >30, and between 90 and 270, respectively.

Landslide occurrence shows clearly correlation with the topographic conditions. At all the three sites, over 50% landslides occurred in the derived roughness, aspect and hillslope areas over the threshold values, respectively (Table 4). In order to evaluate the actual correlation between the above topographic parameters and landslide occurrence, we apply equal landslide density as the main factor. Equal landslide density is the ratio between the landslide areas and the corresponding areas derived by applying the roughness, hillslope and slope aspect thresholds. Among the three parameters, roughness is closely correlated to landslide rather than hillslope, i.e., the highest equal area landslide density. The intersection regions are derived from the intersection regions of the roughness, hillslope and slope aspect areas by applying the above thresholds. Therefore, the areas decreased significantly and the landslides occurred in the intersection region is lower than 40%. However, the intersection region is only 20–69% of the areas derived from roughness, hillslope, and slope aspect map. Consequently, the equal area density of the co-seismic landslide is still the highest. The geological structure of Longmen Shan Thrust Belt and seismic shake information of PGA data both suggest that the co-seismic landslides mainly occurred in regions within the up-down and horizontal PGA contour of 150 and 200 gal on the hanging wall side (Figure 6).

Post-earthquake DEM analysis indicates the medium roughness and hillslope regions are becoming rougher and steeper after the Wenchuan earthquake. The roughest relief and steepest slopes are smoothed by the Wenchuan earthquake (Figure 5). This indicates that the medium topographic roughness and hillslope are modified by the Wenchuan-like strong earthquakes or landslides. However, slope aspect did not change much, which indicates the formation and modification of slope aspect is not directly related to single tectonic events such as strong earthquake or landslide. The roughest and steepest regions are co-seismically smoothed by the Wenchuan earthquake. Thus, rough and steep regions are difficult to stand for a long geological epoch with repeated strong earthquakes. Previous studies have demonstrated that landslides have played an important role in the surface processes in Longmen Shan region (Meng et al. 2006). Consequently, our results indicate that the co-seismic landslides are controlled both by topographic conditions and ground motion. The strong earthquakes play an important role in local topographic formation and modification by triggering co-seismic landslides.

## Conclusions

Based on the data and analyses of the present study, we arrived at the following conclusions.

1. Collaborative topographic analyses are efficient in landslide susceptibility evaluation. The co-seismic landslides are related to the topographic roughness, hillslope and slope aspect. The topographic thresholds are >1.2, between 90 and 270, and 30, respectively. The co-seismic landslides are also directly related to PGA values, which is usually occurred on hanging wall within the up-down and horizontal PGA contour of 150 and 200 gal, respectively.
2. Topographic conditions such as roughness and hillslope are controlled by tectonic event like the Wenchuan earthquake, however, slope aspect is not modified by such event.
3. The roughest relief and steepest slope regions were smoothed after the Wenchuan earthquake, however the medium roughness and slope regions became rougher and steeper, respectively.

## Authors’ original submitted files for images

Below are the links to the authors’ original submitted files for images. Authors’ original file for figure 1 Authors’ original file for figure 2 Authors’ original file for figure 3 Authors’ original file for figure 4 Authors’ original file for figure 5 Authors’ original file for figure 6
