# Supplementary figures and images for: Co-seismic landslide topographic analysis based on multi-temporal DEM—A case study of the Wenchuan earthquake
Source: Springerplus. 2013 Oct 17;2(1):544. doi: 10.1186/2193-1801-2-544 (PMC3806981; doi:10.1186/2193-1801-2-544)

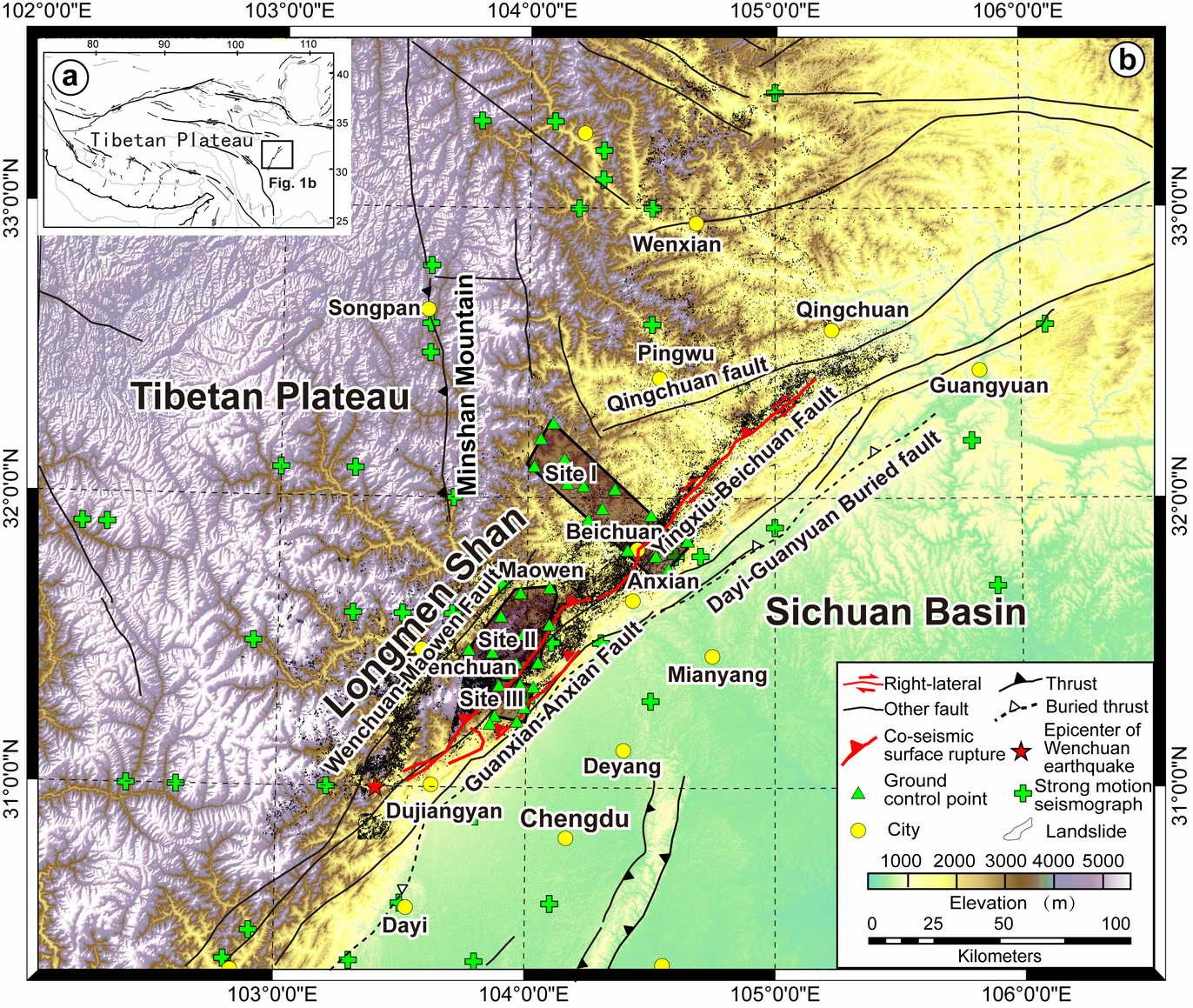

Supplement: Supplementary file 1 — Authors’ original file for figure 1 [file 40064_2013_592_MOESM1_ESM.jpeg]

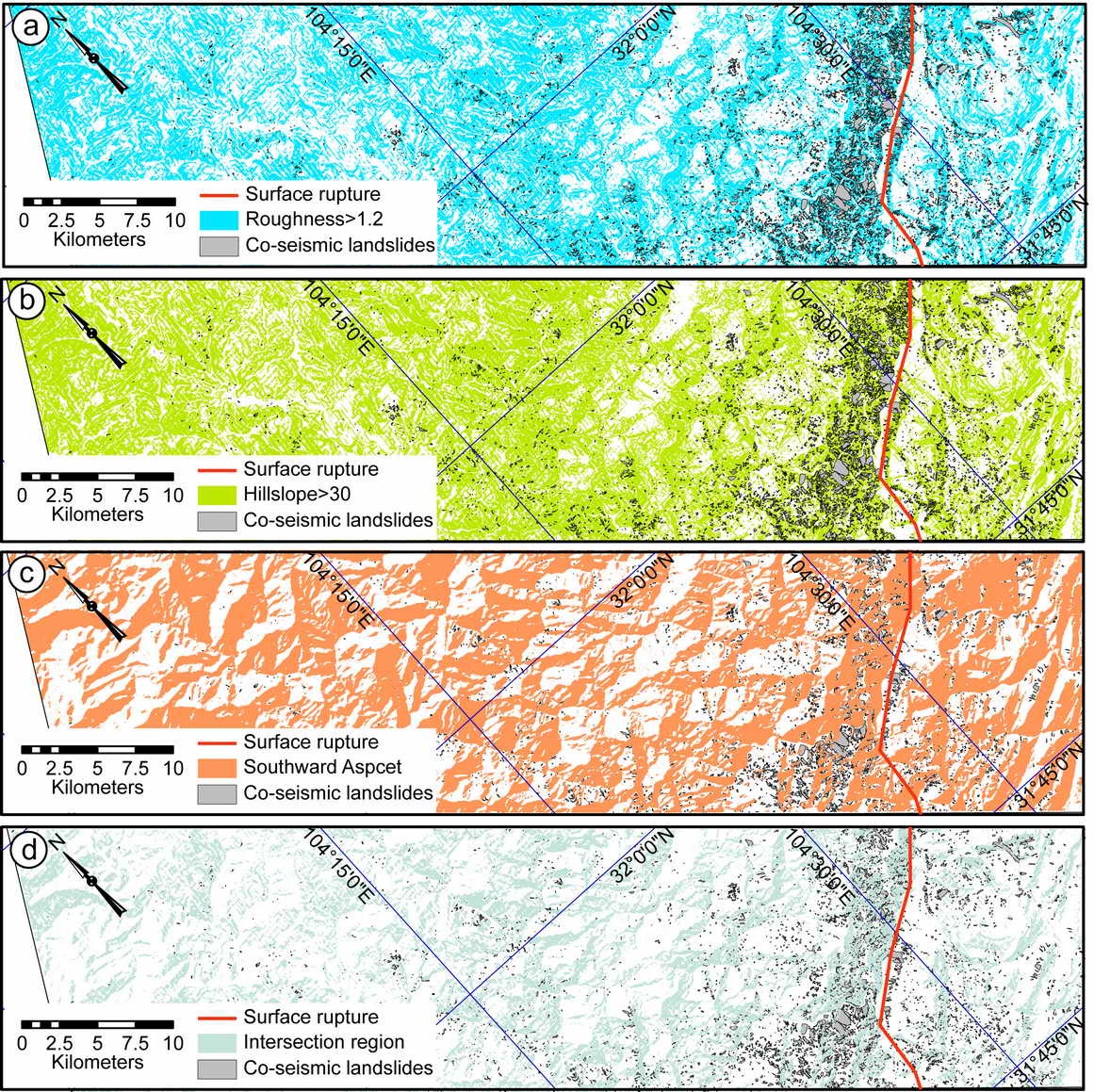

Supplement: Supplementary file 2 — Authors’ original file for figure 2 [file 40064_2013_592_MOESM2_ESM.jpeg]

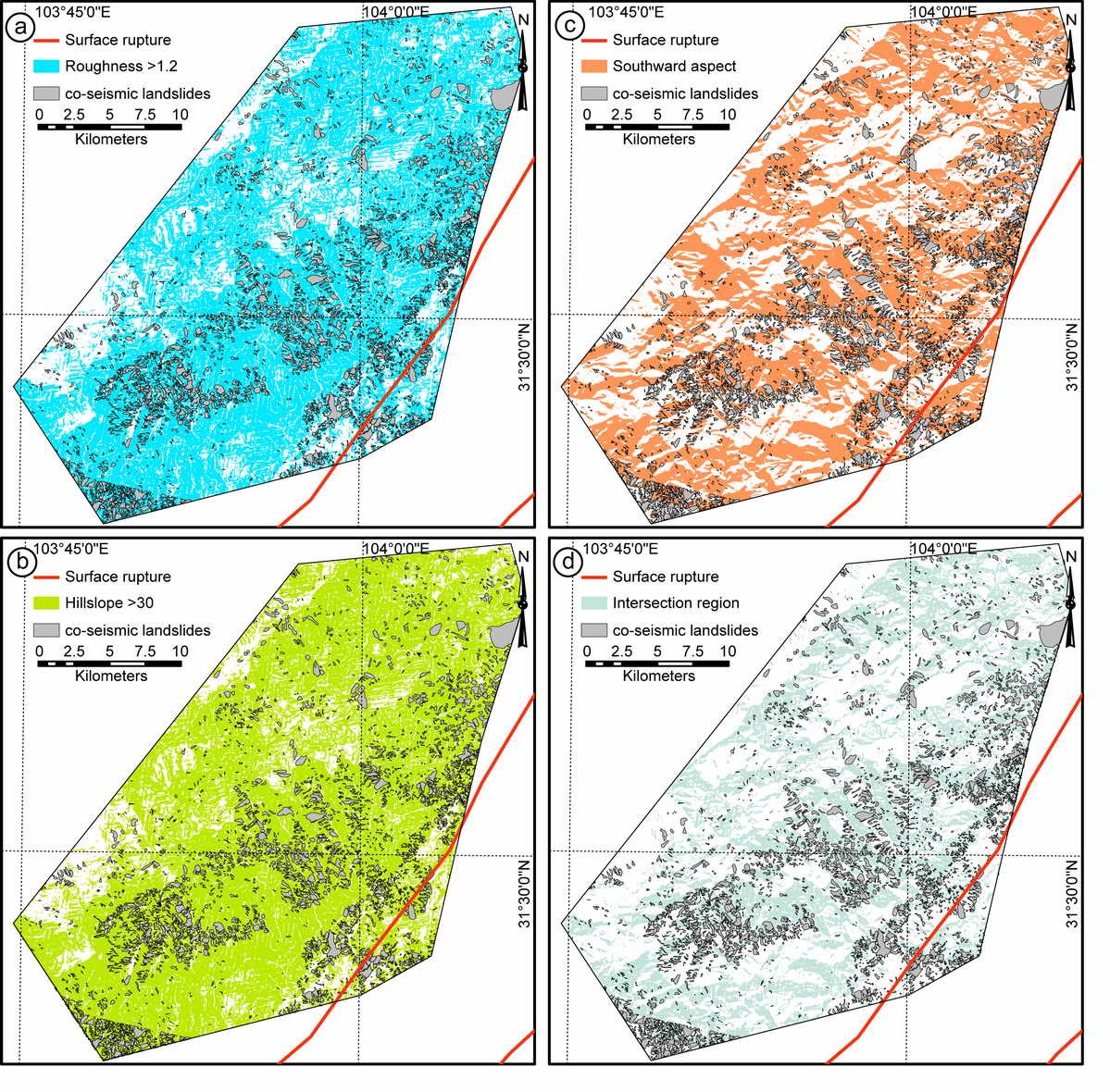

Supplement: Supplementary file 3 — Authors’ original file for figure 3 [file 40064_2013_592_MOESM3_ESM.jpeg]

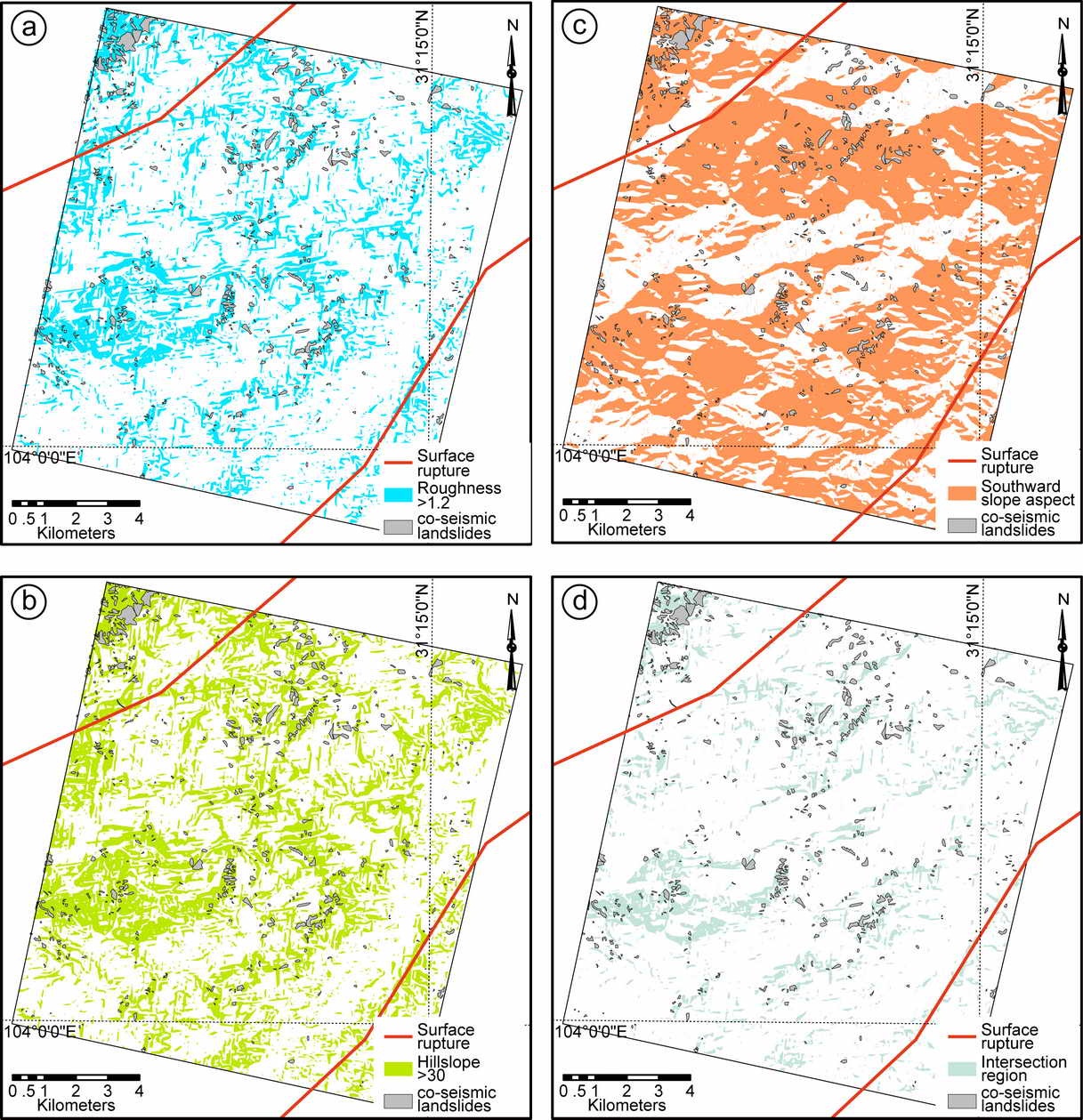

Supplement: Supplementary file 4 — Authors’ original file for figure 4 [file 40064_2013_592_MOESM4_ESM.jpeg]

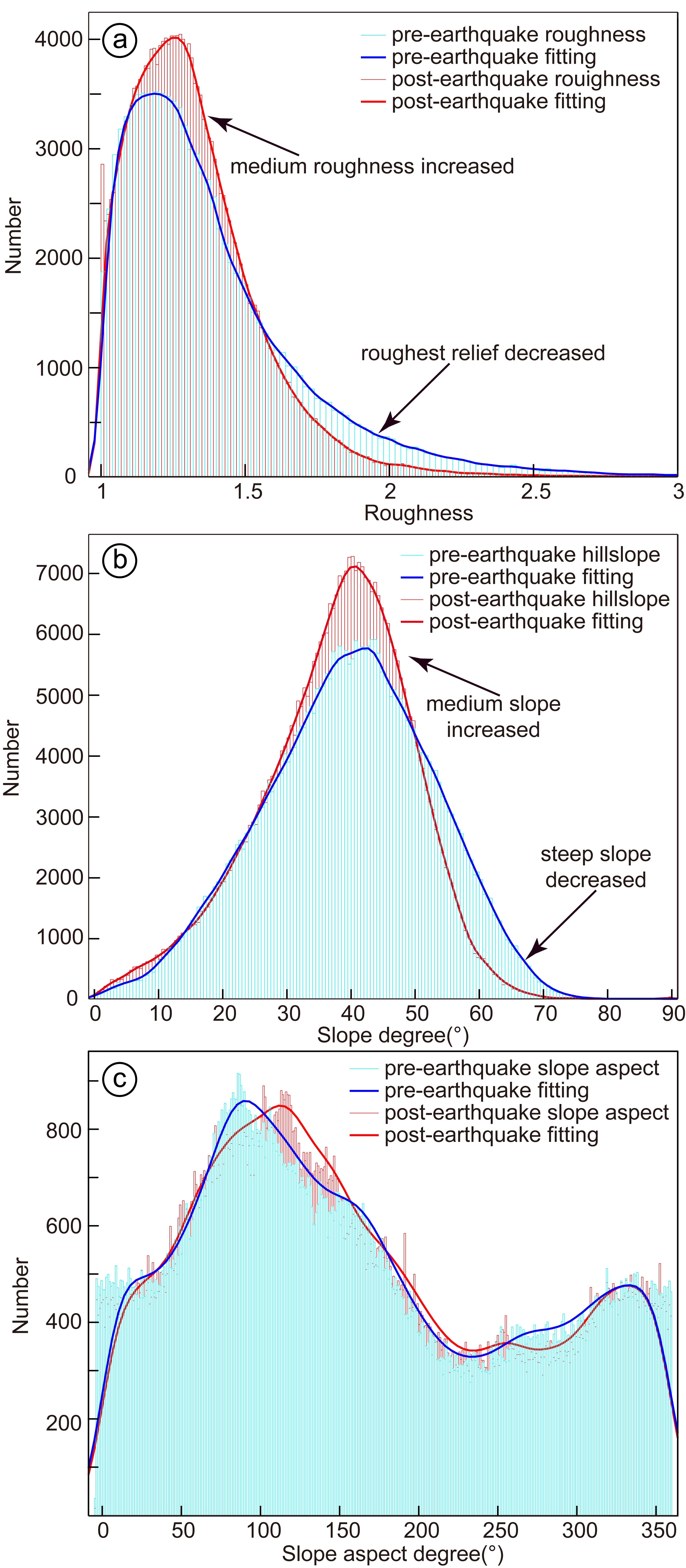

Supplement: Supplementary file 5 — Authors’ original file for figure 5 [file 40064_2013_592_MOESM5_ESM.jpeg]

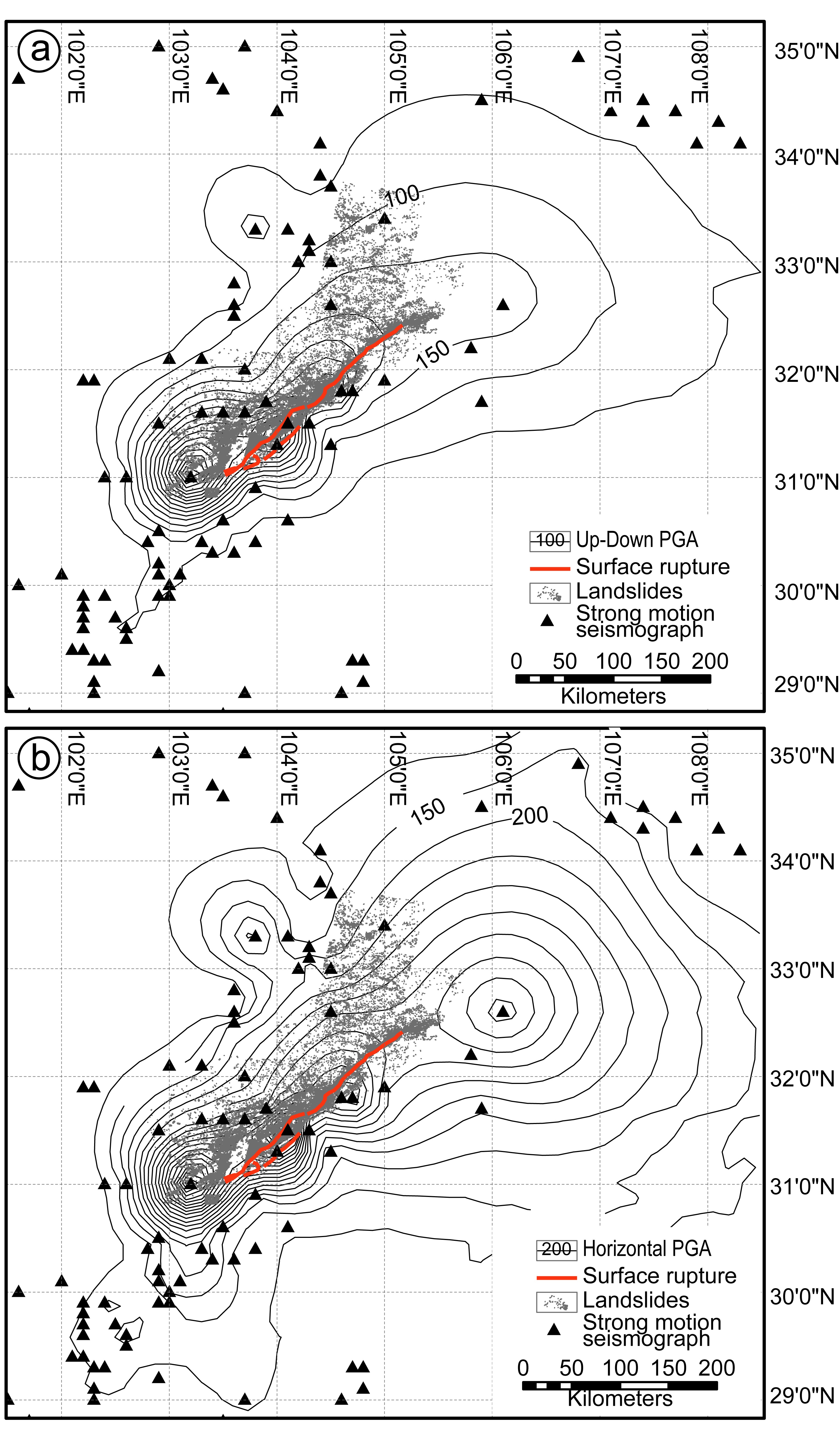

Supplement: Supplementary file 6 — Authors’ original file for figure 6 [file 40064_2013_592_MOESM6_ESM.jpeg]
